# Determination of efflux activity in Enterococci by Hoechst accumulation assay and the role of zinc oxide nanoparticles in inhibition of this activity

**DOI:** 10.1186/s12866-022-02595-x

**Published:** 2022-08-09

**Authors:** Mohammad Hossein Sobhanipoor, Roya Ahmadrajabi, Hossein Hosseini Nave, Fereshteh Saffari

**Affiliations:** 1 Department of Medical Microbiology (Bacteriology and Virology), Afzalipour Faculty of Medicine, Kerman University of Medical Sciences, Kerman, Iran; 2 Medical Mycology and Bacteriology Research Center, Kerman University of Medical Sciences, Kerman, Iran; 3 Department of Microbiology and Virology, Kerman University of Medical Sciences, 22 Bahman Blvd, Kerman, Iran

**Keywords:** *Enterococcus*, Efflux pump, Hoechst, Nanoparticles, Zinc oxide, H33258 accumulation assay, Efflux inhibitor

## Abstract

**Background:**

Contribution of efflux pumps in development of antimicrobial resistance has been largely addressed in Gram negative and to a much lesser extent in Gram positive bacteria. Measuring accumulation of Hoechst (H) dye is known as a safe and rapid method for monitoring efflux activity in bacteria. Antimicrobial effects of metal nanoparticles have been attributed in part to inhibition of efflux pumps. This study aimed to first determine efflux activity in enterococci by Hoechst accumulation assay, and to second characterize the role of zinc oxide nanoparticles (ZnONPs) in inhibition of these pumps.

**Results:**

Increased accumulation of Hoechst dye showed more potential of ZnONPs in efflux inhibition compared with CCCP. H33258 represented more suitability for accumulation studies in enterococci. Two to six-fold reduction in minimum inhibitory concentration (MIC) values of antimicrobial agents in the presence of ZnONPs was observed.

**Conclusions:**

Efflux activity in enterococcal strains can be measured by H33258 accumulation assay. Application of ZnONPs as an efflux inhibitor, may rejuvenate the use of conventional antimicrobial agents against these bacteria.

## Introduction

Extensive use of antibiotics and biocides has increased antimicrobial resistance especially in the healthcare environments, so that currently the emergence of multidrug resistant (MDR) microorganisms is considered as a major therapeutic problem [1].

Efflux pumps are of innate or acquired antimicrobial resistance mechanisms through which bacteria exit harmful compounds such as antibiotics and other chemical substances or even may secrete cellular products. Blocking these pumps can be a promising strategy to restore the susceptibility of resistant strains and improve the efficiency of antimicrobial agents. So, identification of efflux pump inhibitors (EIs) is of interest to many researchers [2].

Among different investigated antimicrobial agents, including chemical compounds or plant- derived substances, the potential of metal nanoparticles (NPs) and their synergistic effects with antimicrobials compounds have been recently proposed [3]. Although not completely understood, these effects have been attributed in part to the efflux inhibitory effects of NPs [3].

For monitoring the activity of efflux pumps in bacteria, several approaches have been developed. One of the robust methods is measurement of Hoechst (H) 33342 (bis-benzamide) accumulation, using fluorometric assay which has been previously adopted for use in Enterobacteriaceae and *Acinetobacter baumannii* [4–6]. This technique is based on the fact that some fluorescent compounds change their wavelength of maximal emission following intercalating with DNA. For Hoechst dyes (H33258, H33342 and H34580), fluorescence increases up to 30 times which is a good signal [7]. This event makes it possible to distinguish between intra- and extracellular localization of the probe in real time. So, these compounds can be applied for assessment of accumulation and efflux activity [4].

Although multidrug resistance efflux pumps have been identified in both Gram positive and Gram negative bacteria, most of the researches have been focused on Gram negatives, such as AcrA/ AcrB/ TolC in Enterobacteriaceae or MexA/MexB/ OprM in *Pseudomonas aeruginosa* [8]. With respect to Gram positives, NorA efflux pump in *Staphylococcus aureus*, has been the forefront of the most studies and the other important pathogens have received less attention [9].

Enterococci are Gram positive cocci which can cause serious problems in hospitalized patients. MDR strains of enterococci are now the leading cause of nosocomial infections worldwide [10]. Using bioinformatics approaches, approximately 23 multidrug transporters have been reported in *Enterococcus faecalis* strains [11]. Among these, involvement of EfrA/B as an ABC transporter efflux pump, and EmeA from major facilitator superfamily (MFS), have been demonstrated in resistance to antibiotics (such as aminoglycosides and fluoroquinolones) and /or reduced susceptibility to biocides [12, 13].

So far, there are some just limited reports on the role of efflux pump inhibitors in Gram positive bacteria and to the best of our knowledge, there is the first report of efflux inhibitory effect of nanoparticles in enterococci. In this study, we aimed to investigate the efflux inhibitory effect of green synthesized zinc oxide (ZnO) NPs in enterococcal isolates by Hoechst accumulation assay. These effects were also tested in few *Escherichia coli* strains. In addition, the effect of AgNPs and CuNPs (commercially available) was tested. In all experiments, carbonyl cyanide-*m*-chlorophenyl hydrazone (CCCP) was applied as a known EI.

## Results

### Bacterial isolates

According to the results, MIC_90_ values for ciprofloxacin and gentamicin were 256 and > 8000 µg/ml, respectively. For biocides, 71% (17/ 24) and 29% (7/24) of isolates showed reduced susceptibility to chlorhexidine digluconate (CHG) and benzalkonium chloride (BCC) respectively. Notably, other than *efr*A/B and *eme*A, no other efflux genes were identified in our isolates. Respectively, *efr*A/B and *eme*A were detected in 100% and 87.5% of isolates. Also, the presence of both genes was detected in 87.5% of isolates (Table 1).

**Table 1:** Distribution of efflux genes among enterococcal isolates according to reduced antimicrobial susceptibility pattern

Isolates (No.)	Antibiotic resistance	Biocide reduced susceptibility	Efflux pump genes
8	CIP, GM	CHG	*efr*A*,efr*B*,eme*A
3	GM	CHG	*efr*A*,efr*B*,eme*A
2	CIP, GM	CHG, BCC	*efr*A*,efr*B*,eme*A
2	CIP, GM	BCC	*efr*A*,efr*B*,eme*A
2	-	CHG	*efr*A*,efr*B*,eme*A
1	-	CHG, BCC	*efr*A*,efr*B*,eme*A
1	-	BCC	*efr*A*,efr*B*,eme*A
1	GM	-	*efr*A*,efr*B*,eme*A
1	CIP	-	*efr*A*,efr*B*,eme*A
1	CIP, GM	BCC	*efr*A*,efr*B
1	CIP, GM	CHG	*efr*A*,efr*B
1	GM	-	*efr*A*,efr*B

*CIP* Ciprofloxacin, *GM* Gentamicin, *CHG* Chlorhexidine digluconate, *BCC* Benzalkonium chloride

### ZnONPs synthesis

**Fig. 1:**
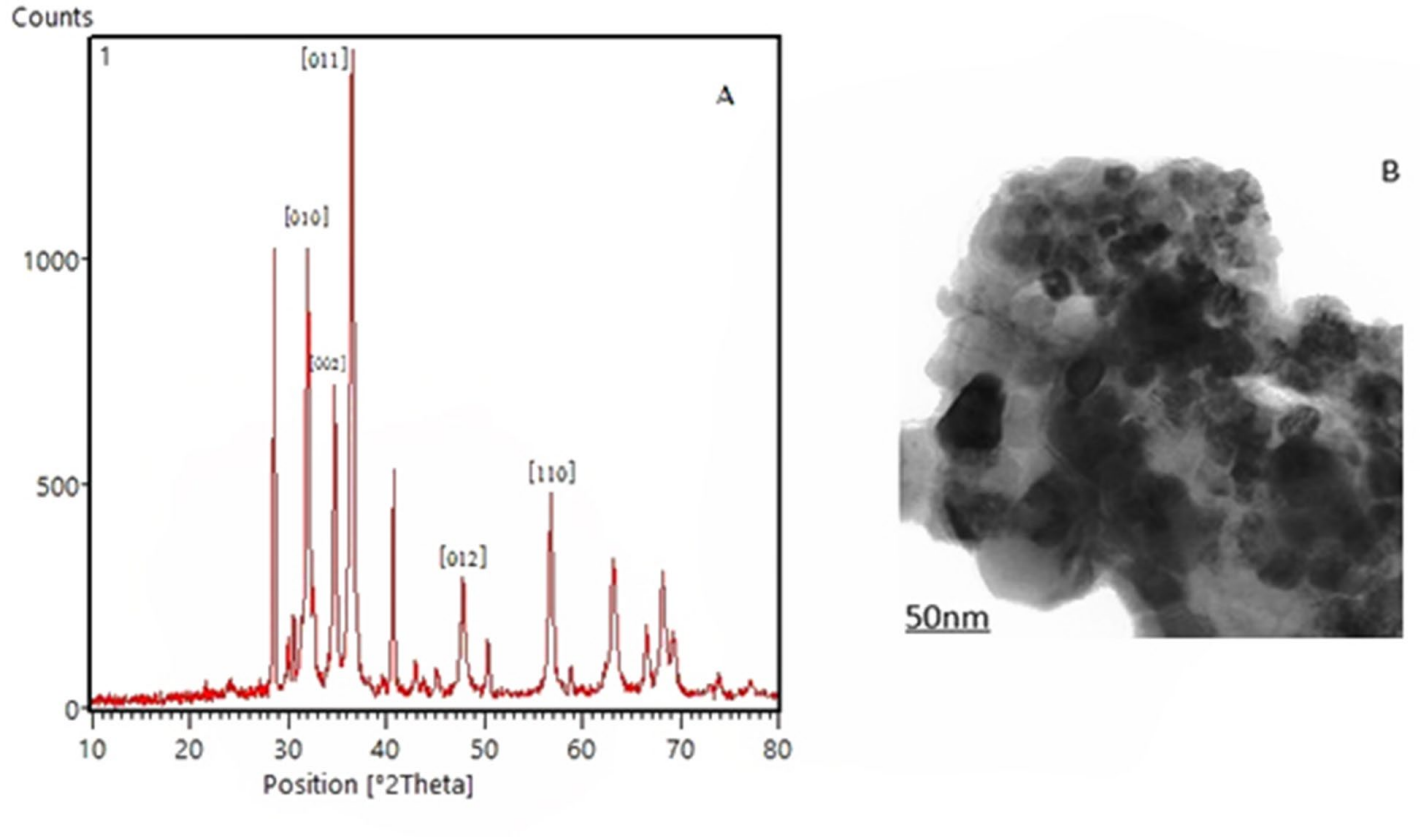
**A** X-ray diffraction pattern of zinc oxide nanoparticles. **B** TEM image of the rectangular shaped zinc oxide nanoparticles

The X-ray diffraction (XRD) analyzed by High Score Plus software (PANalytical B.V Almelo, The Netherlands, version 3.0e) verified the crystal planes of green synthesized ZnONPs (Fig. 1A). In this pattern, peaks appeared at 2θ positions of 31.9, 34.6, 36.4, 47.8, and 56.8°, are related to reflections from crystal planes of (010), (002), (011), (012) and (110), respectively. This pattern is compatible with that previously reported for ZnONPs [14]. Specific and clear peaks affirmed the high purity and crystalline nature of the arranged ZnONPs. In addition,transmission electron microscopy (TEM) examination appeared rectangular morphology of surface appearance of NPs in a range of 10 to 90 nm (Fig. 1B).

### The MIC values of NPs, CCCP and Hoechst toxicity:

The MIC value of CCCP for all isolates was 62.5 µg/ml (except for 2 isolates: 31.25 µg/ml). In all isolates MIC values of ZnONPs and CuNPs, were 8 mg/mL while it was 2 mg/ml for AgNPs. Hoechst dyes at concentration of 2.5 µM showed no significant effect on the viability of bacterial isolates. Also, at sub-MIC concentration of CCCP (25 µg/mL) and NPs, no inhibitory effect on bacterial growth was detected.

### Effect of CCCP on antibiotic resistance or biocide tolerance

To determine the role of efflux pumps in the antibiotic resistance and reduced susceptibility to biocides in 20 enterococcal isolates, we evaluated and compared the MICs of antimicrobial agents in the presence (25 μg/mL) and absence of CCCP. The results showed that in 70% (14/ 20) of gentamicin -resistant isolates, gentamicin MIC was decreased at least four folds in the presence of efflux pump inhibitor. Similarly, in 70% (14/ 20), 40% (8/ 20), and 30% (6/ 20) of isolates, antimicrobial susceptibility increased at least four folds to CIP, CHX and BCC in the presence of efflux pump inhibitor, respectively (Table 2). This confirms that efflux pump can be involved in extrusion of related antimicrobial agents. In following, accumulation assay was conducted.

**Table 2:** Effect of CCCP on fold reduction in MICs of antimicrobial agents in enterococcal isolates

Fold reduction (F) in MIC	Number of isolates with fold reduction in MIC			
	GM	CIP	CHG	BCC
14F	1	-	-	-
10F	2	-	-	-
8F	4	2	-	-
6F	3	5	2	-
4F	4	7	6	6

*CIP* Ciprofloxacin, *GM* Gentamicin, *CHG* Chlorhexidine digluconate, *BCC* Benzalkonium chloride

### Determination of the most effective concentrations of NPs and CCCP for accumulation assay:

Monitoring the accumulation of Hoechst dyes in the presence of different concentrations of NPs and CCCP, was resulted to find concentrations of NPs and CCCP in which maximum level of fluorescence was reached. According to our findings, concentrations of 160 µg/ml for ZnONPs, and 40 µg/ml for Ag and CuNPs were determined for accumulation assay in enterococci. These concentrations for assessment in *E. coli* isolates were 40 µg/ml (for AgNPs) and 80 µg/ml (for ZnO and CuNPs). Also, the used concentrations of CCCP and Hoechst dyes were 25 µM and 2.5 µM, respectively.

### Hoechst accumulation assay

Hoechst is a fluorescent probe which is widely used to indicate the efflux pumps activity in bacteria. In this study, both H33258 and H33342 were tested for accumulation assay, but in enterococci, the best result was derived from H33258 vs H33342 in *E. coli* strains (data not shown).

**Fig. 2:**
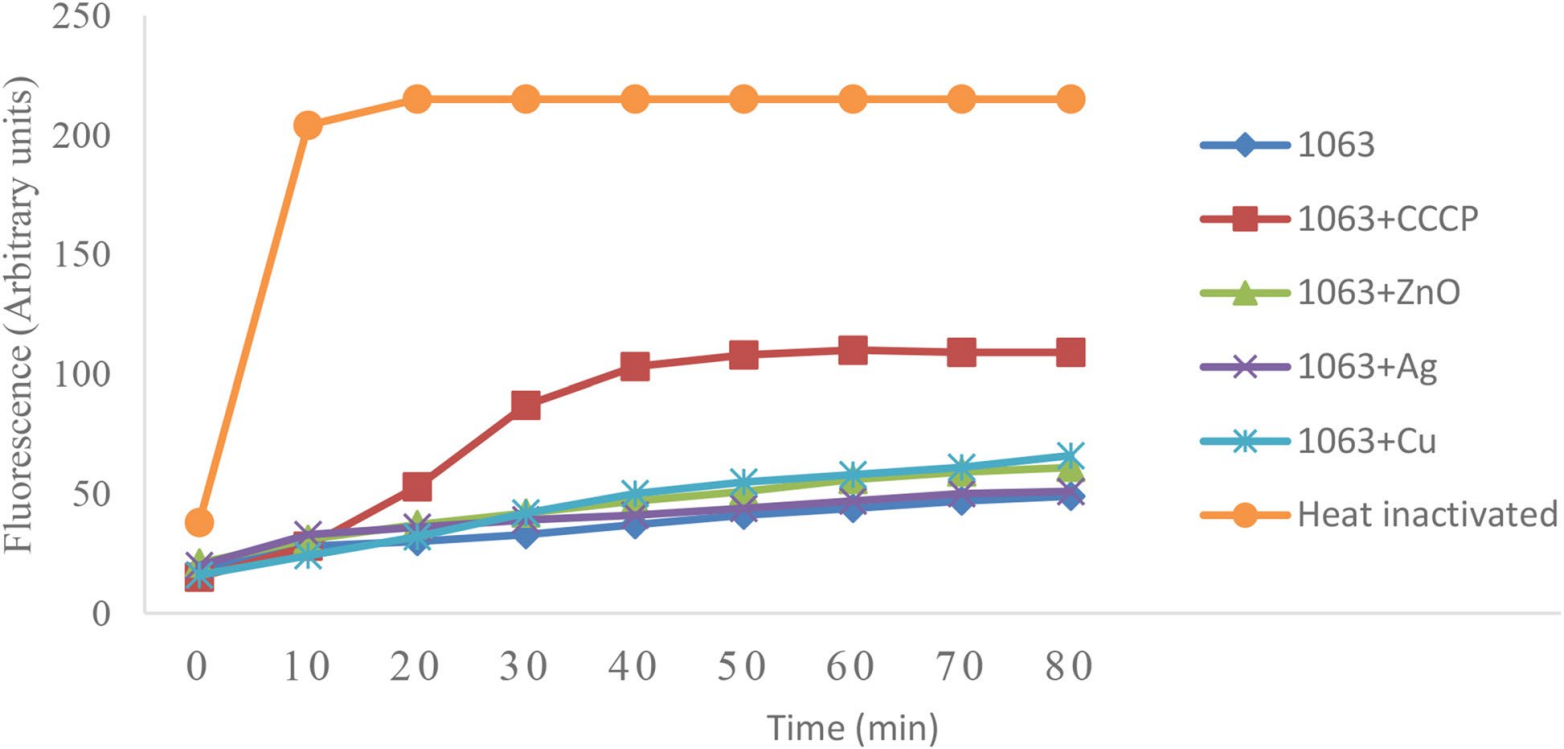
Hoechst H33342 accumulation assay in *E.coli* isolate (#1063) in the presence and absence of CCCP and different nanoparticles (Ag, CU and ZnO). Heat-inactivated bacteria served as a positive control. The fluorescence intensity was recorded at excitation and emission wavelengths of 360 nm and 460 nm, respectively, over 80 min incubation period

The effect of the efflux pump inhibitor (CCCP) and nanoparticles (ZnONP, AgNP and CuNP) on efflux pumps was evaluated by monitoring H33342 accumulation in *E*. *coli* strains. Heat-inactivated bacteria that served as a positive control accumulated maximal levels of H33342, rapidly (Fig. 2). None of the NPs could not significantly increase the accumulation of H33342 in *E. coli* isolates, compared to untreated ones (Fig. 2).

**Fig. 3:**
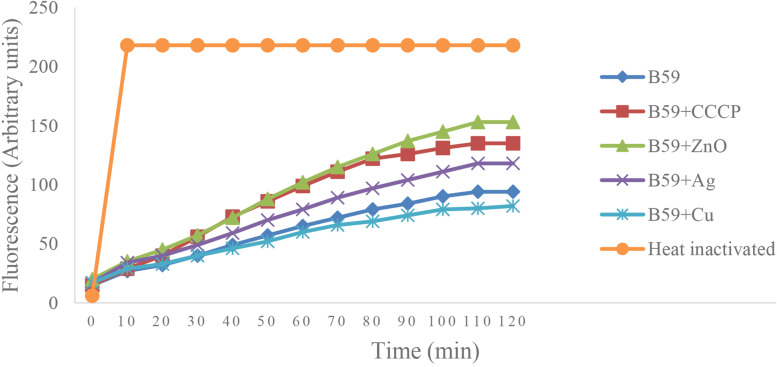
Hoechst H33258 accumulation in enterococcal isolate (#B59) in the presence and absence of CCCP and different nanoparticles (Ag, CU and ZnO). The fluorescence intensity was recorded at excitation and emission wavelengths of 360 nm and 460 nm, respectively, over 120 min incubation period

Similarly, in enterococci, the presence of CuNPs did not increase the accumulation of H33258 compared to untreated strain. Although AgNPs were effective, the increase in fluorescence levels was lower than those in the presence of CCCP or ZnONPs. The steady-state level of H33258 was significantly higher in isolates treated with CCCP or ZnONP when compared with the untreated strain, suggesting an increased level of efflux (Fig. 3).

The fold change in fluorescence in the steady-state phase in the presence and absence of CCCP and ZnONPs was calculated for each isolate. Addition of CCCP and ZnONPs caused a significant increase (*P* ≤ 0.05) in the level of H33258 accumulated at steady-state for most enterococcal isolates.

**Fig. 4:**
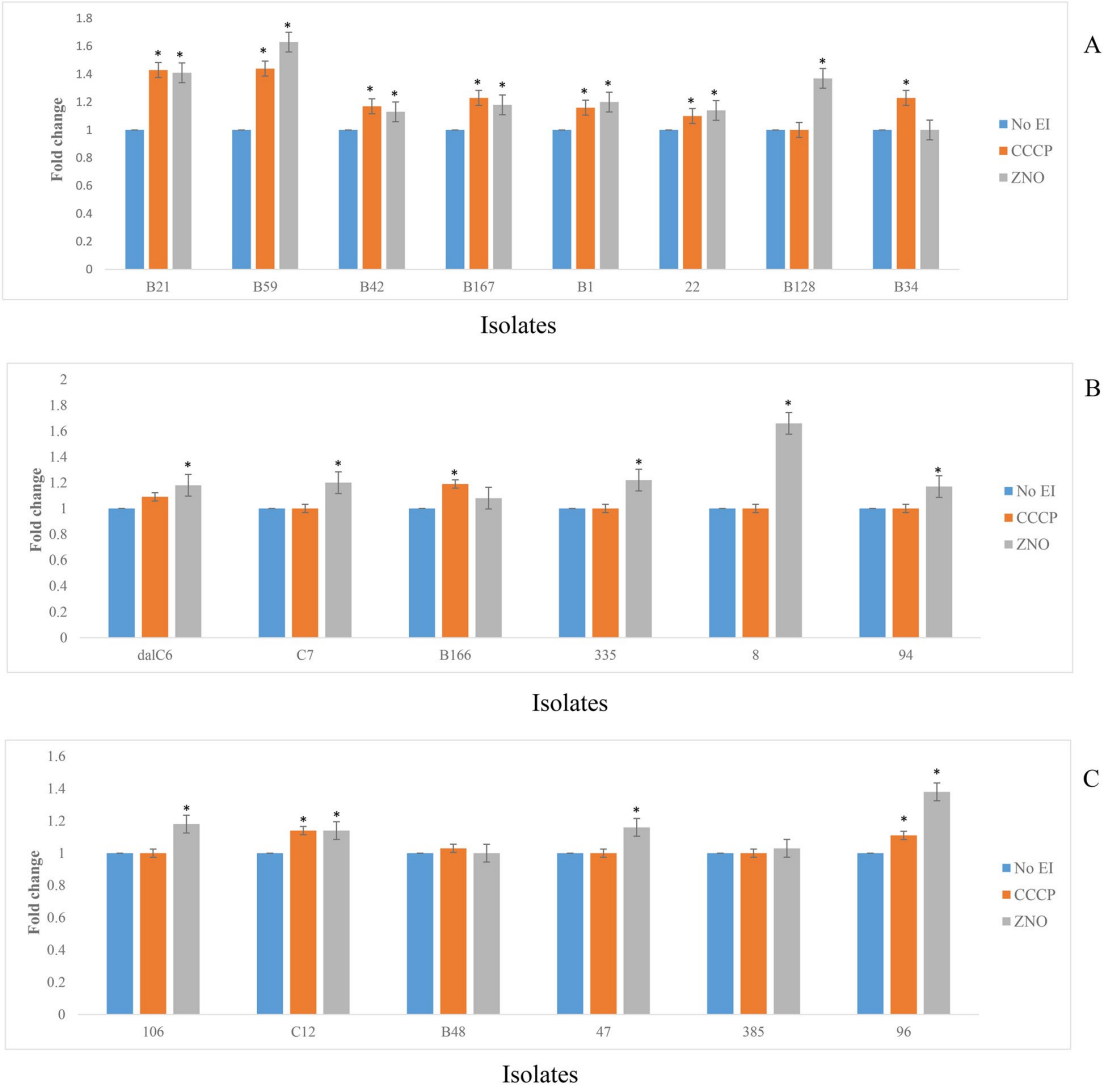
**A, B, C** Fold differences in levels of H33258 accumulated by enterococcal isolates at steady- state with or without efflux inhibitors (CCCP and ZnONPs). (*) Significant difference in dye accumulation in the presence of efflux inhibitors in comparison with the lack of them. *P*-value of ≤ 0.05 was considered significant. Each experiment was repeated three times

According to results, in 80% (16/20) of isolates, accumulation of H33258 increased significantly (*P* < 0.05) in the presence of ZnONPs. Of these, in 8 isolates, increased accumulation was also observed in the presence of CCCP and in eight remaining ones, only ZnONPs were able to increase dye accumulation, significantly. Conversely, in 10% of isolates (2/20), only the effect of CCCP was significant. The largest fold changes were1.44 & 1.63 for CCCP and ZnONP, respectively. Notably, in two isolates, no significant effect of CCCP or NPs was detected (Fig. 4).

### Effect of ZnONPs on antibiotic resistance or biocide tolerance

The susceptibility of 10 enterococcal isolates that were resistant or showed reduced susceptibility to CIP, GM and CHG was tested in the presence of ZnONPs. Results showed anti efflux activity (at least twofold reduction in MIC) in the presence of ZnONPs. The effect of ZnONPs on the MIC values of antimicrobial substances is presented in Table 3. According to results, ZnONPs caused 2 to 6 fold reduction in MIC values of antimicrobial agents.

**Table 3:** Effect of ZnONPs on MICs of antimicrobial agents in enterococcal isolates

Isolates	Fold reduction in MIC		
	CIP	GM	CHG
*E. faecalis* B1	4	4	2
*E. faecalis* B128	0	2	6
*E. faecalis* B166	4	6	2
*E. faecalis* B34	0	0	2
*E. faecalis* B48	2	0	2
*E. faecalis* 47	2	2	0
*E. faecalis* B59	2	0	2
*E. faecalis* 385	0	2	2
*E. faecalis* B21	2	0	2
*E. faecium* 22	4	4	2

*CIP* Ciprofloxacin, *GM* Gentamicin, *CHG* Chlorhexidine digluconate

## Discussion

Over expression of efflux pumps is one of the defenses which bacteria have evolved against antimicrobial agents. These pumps may be selective for one substrate or transport a broad range of compounds (may be structurally unrelated) [8]. Bypassing these pumps, is one of the important tools to combat MDR strains. Our findings showed that the efflux of H33258 was inhibited by ZnONPs in 80% of isolates, of which only 50% (ten isolates), were also affected by CCCP. This discrepancy, may be attributed to differences in action mechanism of ZnONPs and CCCP. The other explanation is that the presence of different efflux pumps which Hoechst dyes are substrates for them, may be strain specific.

Generally, efflux inhibition can be achieved by different approaches: 1) reducing the affinity of substrate (drugs) to binding site of efflux pumps; 2) increasing the cell-permeability of substrates; 3) down-regulating the expression of efflux pumps; 4) falling down the energy required for efflux (as known for CCCP); 5) inhibiting the assembly of efflux components; 6) blocking the efflux pumps by a designed molecular plug; 7) creating a competition between substrate and antimicrobial agents [8].

For NPs, two possible mechanisms have been described as aforementioned in points 6 and 7 [3]. However, the exact mechanism has not been completely defined. In review studies by Alavi et al. [15, 16] on antibacterial properties of NPs, various mechanisms were pointed out, including increase in the permeability of antibiotics into the cells, pore creation and membrane destruction, prevention of biofilm formation, inactivation of electron transfer chain, production of reactive oxygen species (ROS), denaturation of macromolecules, and effect on cellular signals. Taken together, more potential of ZnONPs than CCCP in dye accumulation, suggests that it can be a suitable candidate for anti-efflux studies in future. The similar issue has been reported by Banoee et al. who mentioned the interruption activity of ZnoNPs in NorA protein in *S. aureus* [17]. Also, the anti-efflux activity of green synthesized AgNPs against MDR strains of *A. baumannii* and *P. aeruginosa,* has been described previously [18].

In this study, to achieve the best results in accumulation assay, two Hoechst dyes, H33342 and H33258 were applied. Although applications of theses dyes have been demonstrated to be similar, H33342 show tenfold more cell-permeability than H33258 [7, 19]. In studies by Coldham et al. [4] and Richmond et al. [5], H33342 accumulation assay was described for Enterobacteriaceae and *A. baumannii*. In our study, H33258 represented to be more suitable option for accumulation studies in enterococci. This can be related to differences in cell wall structure of Gram positive and Gram negative bacteria. For explanation, it can be said that the release of large amounts of accumulated H33342 inside the Gram positive cells is not considerable and subsequently not measurable in comparison to H33258. Although proving this issue requires further studies, disparity between data acquired with these two dyes emphasizes the need to use more than one substrate for measurement the efflux activity.

Another point is that, in spite of the other researches which have been frequently performed on one strain and its mutant, in this study, 20 enterococcal isolates were investigated. So, acquirement the non- identical results in this study, demonstrates that efflux power may be strain specific. Also, there were some differences between the effects of ZnONPs on the efflux activity compared to MIC. This can be justified as clinical isolates possess different antimicrobial resistance genes which may mask the effect of efflux genes.

In conclusion, this study showed the potential of ZnONPs for efflux inhibition in enterococci and demonstrated that H33258 can be applied for evaluation the contribution of efflux pumps in these organisms.

## Materials and methods

### Bacterial strains

At first, antimicrobial activity of enterococcal isolates (n = 100) was determined against ciprofloxacin, gentamicin (Exir pharmaceutical Co.), CHG (Sigma-Aldrich) and BCC (ACROS Organics) using micro broth dilution method [20]. These isolates were from different specimens of patients and fecal flora of healthy people obtained from bacterial collection at microbiology lab of Kerman University of Medical Sciences. *E. faecalis* ATCC 29212 was used as control strain. PCR experiments were carried out using specific primers for *efr*A/B, *eme*A, *smr*, *oqx*A/B, *qac*A/B, *qac*C, *qac*ΔΕ, *qac*j, *qac*G, *qac*Z to screen efflux associated genes [21–23]. According to the acquired results, finally 24 isolates were selected. These isolates were resistant to ciprofloxacin and /or gentamicin and showed reduced susceptibility to CHG and /or BCC and carried at least one of the efflux associated genes.

### Green synthesis of ZnONPs

ZnONPs were green synthesized from natural sweetener *Stevia rebaudiana* (No. HUEM-14301 provided by Razi University, Kermanshah, Iran.) leaf extract as described by Khatami et al. [14]. In summary, washed and dried leaves were crushed completely. Then, 100 g of Stevia powder was added to 700 ml deionized water and shaked at 160 rpm at room temperature for two days. Following centrifugation at 7000 rpm for 15 min, the supernatant was filtered using Whatman paper No. 1 and mixed with 0.1 mol/ L of zinc acetate solution with stirring at 70-80º C. Finally, blackish green gel was placed in an oven at 90 °C and the sediment was calcined at 600 °C for 2 h. Characterization of the resulted white powder as ZnONPs was assessed using UV–Visible spectrophotometer (Analytic Gena Co.), XRD using Philips Xpert MDP diffractometer equipped with a Cu Kα anode (at Kashan University) and transmission electron microscopy (TEM, FEI Tecnai 20) [14]. AgNPs and CuNPs were purchased from US Research Nanomaterials, Inc.

### Determination the MICs of NPs, CCCP and Hoechst toxicity

Two-fold serial microdilution broth was used to determine the MICs of NPs and CCCP (Sigma Aldrich). For this purpose, concentration ranges of CCCP (0.975–125 µg/ml) and NPs (ZnO, Cu, and Ag) (0.125–16 mg/ml) were used. Toxicity of Hoechst dyes was evaluated by incubating enterococcal isolates at a log growth phase OD of 0.6 (600 nm) for 0, 30, 60, 90, and 120 min and counting the viability of aliquots on blood agar plates [4, 24].

### Determination the MICs of antimicrobial agents in the presence of CCCP

To determine the role of efflux pumps in resistance to tested antimicrobial agents, the MICs of ciprofloxacin, gentamicin, CHG and BCC were determined in the presence of CCCP and compared with those in the absence of inhibitor. More than fourfold reduction in the MIC following the addition of CCCP, confirmed the role of efflux pumps in extrusion the antimicrobial agents [21].

### Hoechst accumulation assays

To investigate the efflux inhibitory potential of NPs, Hoechst accumulation assay was used as described previously [4, 5]. Briefly, bacterial isolates were incubated in Tryptic Soy Broth (TSB) at 37° C overnight. Then, 200 µL of microbial suspension was added to 5 ml TSB and placed in a shaker incubator for 3 to 5 h until the cells reached an OD:0.6 at 600nm (~ 1 × 10^8^ cfu/ml). Following harvesting the cells by centrifugation at 15000 rpm for 15 min, they were re-suspended in 5 ml of sterile PBS and optical density of suspension (at 600 nm) was adjusted to 0.5. Each well of a flat-bottom black plate (Corning, USA) was inoculated by 176 µL of cell suspension along with 4 µL of NPs or CCCP at the required concentration.

The plate was placed inside the plate reader (FLX 800 BIOTEK-USA). Immediately after two readings, Hoechst dye (20 µl) (in this study two Hoechst dyes; H33258 and H33342, were used) was added to the wells at the final required concentration (2.5 µM). Fluorescence was read from the top of the wells using excitation and emission wavelength of 360 nm and 460 nm, respectively. Readings were taken for 60 cycles with a 120 s delay between cycles and a gain multiplier of 120 min. The steady-state accumulation level was defined as a level at which maximum fluorescence was achieved and remained unchanged throughout the time period. Heat-killed bacterial isolate, showing greatest level of fluorescence, was used as positive control.

This experiment was also performed on three ciprofloxacin resistant *E. coli* strains (from our previous study, data not shown) which showed significant reduction (4–6 fold) in MIC to ciprofloxacin in the presence of CCCP.

### Determination the MICs of antimicrobial agents in the presence of ZnONPs

According to the results of accumulation assay, the effect of ZnONPs (2 mg/ml) on the MICs of ciprofloxacin, gentamicin and CHG was determined by micro broth dilution method. Due to sedimentation of NPs and interference in reading the bacterial growth, tetrazolium salts was used [25].

### Statistical analysis

SPSS (IBM SPSS Statistics 22) and Excel (Microsoft) were used to analyze the fluorescence data, drawing the curves and bar graphs. Every experiment was repeated three times. Two-tailed Student's t-test was used to evaluate differences in accumulation between the presence and absence of CCCP and/ or NPs. *P*-value ≤ 0.05 was considered as significant.

## Data Availability

The datasets used and/or analyzed during the current study are available from the corresponding author on reasonable request.

## Abbreviations

ZnONPs: Zinc oxide nanoparticles
H: Hoechst
CIP: Ciprofloxacin
GM: Gentamicin
CHG: Chlorhexidine digluconate
BCC: Benzalkonium chloride
MIC: Minimum Inhibitory Concentration
EI: Efflux Inhibitor
CCCP: Carbonyl cyanide-*m*-chlorophenylhydrazone
XRD: X-ray diffraction
TEM: Transmission Electron Microscopy
PCR: Polymerase Chain Reaction
TSB: Tryptic Soy Broth
